# Dose–response of benazepril on biomarkers of the classical and alternative pathways of the renin–angiotensin–aldosterone system in dogs

**DOI:** 10.1038/s41598-023-29771-x

**Published:** 2023-02-15

**Authors:** Samantha Sotillo, Jessica L. Ward, Emilie Guillot, Oliver Domenig, Lingnan Yuan, Joseph S. Smith, Vojtech Gabriel, Chelsea A. Iennarella-Servantez, Jonathan P. Mochel

**Affiliations:** 1 Department of Veterinary Clinical Sciences, College of Veterinary Medicine, Iowa State University, Ames, IA USA; 2 Ceva Santé Animale, Companion Animal Franchise, Libourne, France; 3 Attoquant Diagnostics, Vienna, Austria; 4 Department of Veterinary Biomedical Sciences, College of Veterinary Medicine, Iowa State University, Ames, IA USA; 5 Department of Large Animal Clinical Sciences, College of Veterinary Medicine, University of Tennessee, Knoxville, TN USA

**Keywords:** Cardiology, Diseases, Medical research

## Abstract

Angiotensin-converting enzyme inhibitors (ACEI) such as benazepril are commonly prescribed in both humans and dogs with heart disease to mitigate the renin–angiotensin–aldosterone system (RAAS); however, the dose-dependent effects of benazepril on comprehensive RAAS components remain unknown. In this study, nine purpose-bred healthy dogs received three different dosages of oral benazepril (0.125 mg/kg, 0.25 mg/kg, or 0.5 mg/kg) in a randomized crossover design following induction of RAAS activation by consuming a low-sodium diet. Blood samples were collected at serial time intervals after benazepril dosing to measure plasma benazeprilat (active metabolite of benazepril) and serum RAAS biomarkers. Blood pressure and echocardiogram were performed at baseline and after each benazepril administration. Time-weighted averages for RAAS biomarkers for 12 h post-dose and hemodynamic variables were compared between dosing groups using Wilcoxon rank-sum testing. Compared to the lowest dosage of benazepril (0.125 mg/kg), the highest dosage (0.5 mg/kg) resulted in lower time-weighted average values of angiotensin (Ang) II (− 38%, P = 0.004), Ang1-5 (− 53%, P = 0.001), ACE-S (surrogate for ACE activity; − 59%, P = 0.0002), and ALT-S (surrogate for alternative RAAS activity; − 22%, P = 0.004), and higher values of AngI (+ 78%, P = 0.014) and PRA-S (surrogate for plasma renin activity; + 58%, P = 0.040). There were no relevant differences between dosing groups for blood pressure or echocardiographic variables. Knowledge of dose-dependent alterations in biomarkers of the classical and alternative RAAS pathways could help inform clinical trials for dosage optimization in both dogs and humans.

## Introduction

Congestive heart failure (CHF) is a common cause of morbidity and mortality in both humans and domestic dogs^1,2^. A mainstay of treatment for CHF in both species is angiotensin-converting enzyme inhibitors (ACEI) such as benazepril^2–6^. Angiotensin-converting enzyme inhibitors mitigate the renin–angiotensin–aldosterone system (RAAS), a neurohormonal system that becomes activated in states of decreased renal blood flow^7–9^ and functions to preserve intravascular volume and perfusion pressure in situations of decreased cardiac output. Mechanisms and consequences of RAAS activation are similar between humans and dogs: chronic activation of the RAAS is both caused by and contributes to progression of heart disease^9^. Given the similarity between dogs and humans in terms of the pathophysiologic implications of RAAS in heart disease, RAAS-mitigating therapies are an intriguing target for a translational One Health-based approach to dosage optimization.

**Figure 1:**
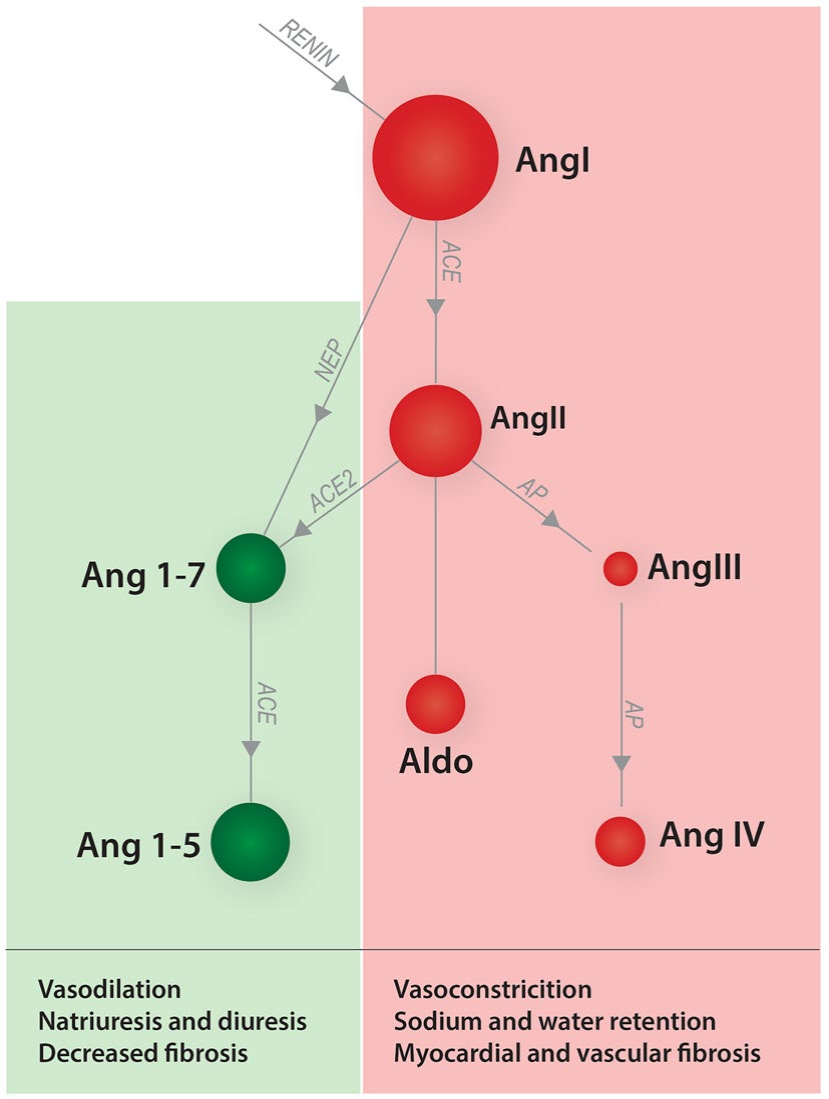
Simplified schematic representation of the classical (red shading) and alternative (green shading) arms of the renin–angiotensin–aldosterone system. Red shading indicates activity of the classical renin–angiotensin–aldosterone system (RAAS); green shading indicates activity of the alternative RAAS. The sizes of the circles are proportional to the expected concentration of each angiotensin peptide in a healthy dog. Arrows indicate enzymes catalyzing conversion of metabolites. *ACE* angiotension converting enzyme, *ACE2* angiotension converting enzyme 2, *Aldo* aldosterone, *Ang* angiotensin, *AP* aminopeptidases, *NEP* neutral endopeptidase.

The classical RAAS pathway (see Fig. 1) refers to the peptide cascade involving the conversion of angiotensin I (AngI) to angiotensin II (AngII) by angiotensin-converting enzyme (ACE), eventually leading to increased adrenal production of aldosterone. Physiologic consequences of AngII and aldosterone include vasoconstriction, sodium and water retention, and myocardial and vascular fibrosis, which are considered maladaptive in the context of CHF^10–12^. Drugs such as ACEI that decrease the production of AngII (and consequently decrease the release of aldosterone) result in balanced vasodilation, reduced sodium and water retention, dilation of the glomerular efferent arteriole, and decreased cardiovascular fibrosis and remodeling^13,14^.

Although ACEI are frequently prescribed in for RAAS modulation and are recommended by consensus panels for the treatment of CHF in both humans and dogs^1,2^, the ideal dosage of ACEI in either species remains unknown. In humans, enalapril is typically prescribed at an initial dose of 2.5 mg twice daily and uptitrated to 10–20 mg twice daily^1,15^. Pharmacokinetic and pharmacodynamic studies comparing various doses of ACEI in healthy dogs have not provided consistent recommendations regarding dosing, with suggestions for optimal dosage ranging from 0.25 mg/kg once daily to 0.5 mg/kg twice daily^16–18^. These studies are limited by the use of plasma ACE activity as the surrogate marker for classical RAAS suppression, which is now considered a suboptimal endpoint to characterize the effects of ACEI on RAAS for several reasons, including ACE-independent mechanisms of AngII and aldosterone production^19–22^ and the potential for renin activation and accumulation of AngI during ACEI therapy^23^. Pharmacodynamic results are also complicated by the chronobiology of RAAS activity^9,24,25^, such that the efficacy of once daily ACEI dosing might vary with respect to time of dosing and feeding^26^.

In terms of hemodynamic benefit, laboratory studies of dogs with experimentally-induced heart or kidney disease have demonstrated reduced systemic arterial blood pressure (BP), decreased mitral regurgitation fraction, and decreased left atrial pressure following ACEI administration at various doses^27–31^. A dose-dependent effect on BP reduction has been demonstrated in cats receiving either enalapril^32^ or benazepril^33^, but dose-dependent hemodynamic effects of ACEI have not been reported in dogs.

Beyond questions of pharmacokinetics and pharmacodynamics, the degree and duration of classical RAAS suppression needed to achieve *clinical* benefits of vasodilation and long-term cardioprotection remain unknown. Clinical trials of dogs with naturally-occurring cardiovascular disease have shown inconsistent long-term benefit utilizing dosages of ACEI ranging from 0.25 to 1.0 mg/kg/day^3,34–38^. While previous studies have assessed the effect of various ACEI doses in canine heart disease, relatively few have compared ACEI dosages *within* the same study, and cross-study comparisons are inherently difficult. A recent retrospective study suggested that twice daily dosing of ACEI was associated with improved long-term outcome in dogs with naturally-occurring cardiovascular disease compared to once daily dosing^39^, but these findings have yet to be confirmed prospectively.

Furthermore, while the classical pathway has been the historical focus of RAAS-mitigating drug therapy in both human and veterinary medicine, it is now well-recognized that the RAAS homeostatic mechanism also includes additional signaling pathways that balance the effects of the classical RAAS. Specifically, the alternative RAAS pathway (see Fig. 1) involves the conversion of AngII by the enzyme angiotensin-convertiny enzyme 2 (ACE2) into angiotensin 1–7 (Ang1-7), with downstream signaling leading to vasodilation, diuresis, natriuresis, and mitigation of vascular inflammation^40^. Therefore, the alternative RAAS pathway provides an internal counterregulatory mechanism that can partly mitigate the negative effects of AngII and aldosterone. In theory, the ideal RAAS-modulating therapy would downregulate the classical RAAS and upregulate the alternative RAAS pathway^41^. However, little information exists currently regarding the effects of ACEI on the alternative RAAS pathway in dogs^42^, and no previous studies have investigated alternative RAAS endpoints for dosage optimization. Accumulating data regarding the effects of ACEI on a comprehensive profile of classical and alternative RAAS biomarkers in dogs would provide valuable translational information for similar dosage optimization of ACEI in humans.

The purpose of the present study was to determine the dose–response relationship between oral benazepril dosing and (1) biomarkers of RAAS activity (including both classical and alternative RAAS biomarkers, as assessed by a comprehensive RAAS fingerprint) and (2) hemodynamic parameters (BP and echocardiography). We hypothesized that compared to lower dosages of benazepril, higher dosages would be associated with lower levels of biomarkers associated with the classical RAAS pathway (i.e. AngII and aldosterone), higher levels of biomarkers associated with the alternative RAAS pathway (i.e. Ang1-7, Ang1-5), lower BP, and lower echocardiographically-estimated left atrial pressure.

## Materials and methods

### Animals

Experimental procedures were approved by the Institutional Animal Care and Use Committee at Iowa State University (protocol number 19-344). All methods were performed in accordance with the relevant guidelines and regulations. All methods are reported in accordance with ARRIVE guidelines.

Nine purpose-bred beagles (5 castrated males and 4 spayed females, 40–42 months old) were randomized into three treatment groups based on body weight and sex using a partial crossover (ABC/BCA/CAB) design. Dogs were members of a colony owned by Iowa State University Laboratory Animal Resources. Systemic and cardiovascular health of all dogs was assessed prior to the study (on day − 5, pre-dose) via physical examination, routine laboratory screening (complete blood count, serum biochemical analysis), BP measurement, and echocardiography. The dogs were pair housed in the Laboratory Animal Resources unit at the Iowa State University College of Veterinary Medicine. Housing conditions were standardized with ambient temperatures of 18 °C, a 12-h light cycle (07:00 to 19:00), and access to water ad libitum.

### Study design

**Figure 2:**
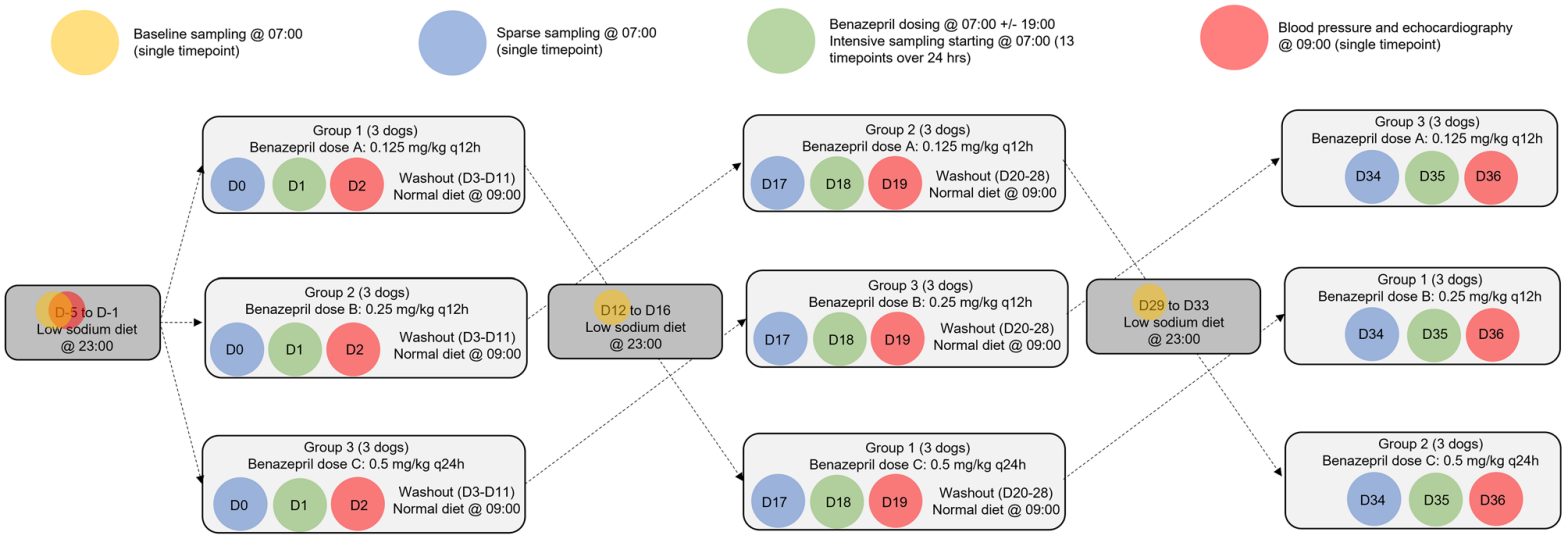
Schematic representation of crossover study design and timeline for sample collection. There are three experimental cycles (treatment periods) during which randomized groups of 3 dogs cross over to receive each of 3 benazepril doses (0.125 mg/kg twice daily, 0.25 mg/kg twice daily, and 0.5 mg/kg once daily). Prior to each cycle, there is a 5-day period of RAAS activation (by consuming a low-sodium diet); between cycles, there is a 10-day washout period during which dogs consume their normal diet. Diagnostic testing performed at each timepoint is indicated by color-coded shaded circles. See text for further details. D, day of the study period; q12h, twice daily; q24h, once daily.

This 35-day prospective study was divided into three cycles with three different oral benazepril dosing groups of 0.125 mg/kg twice daily, 0.25 mg/kg twice daily, and 0.5 mg/kg once daily (see Fig. 2 for graphical representation of study design). These dosages were chosen to optimize the opportunity for mathematical modeling of theoretical dosages that were not directly investigated and of steady-state following repeated dosing. Dogs were sampled in the same order at each time point, and the exact sampling time was recorded. Blood sample collection was divided into baseline sampling days (days -5, 12, and 29), sparse sampling days (days 0, 17, and 34), and intensive sampling days (days 1, 18, and 35). Baseline and sparse sampling occurred at 07:00. After sample collection on baseline sampling days, dogs were fed a low-sodium diet [Hill’s Prescription Diet h/d Heart Care; 17 mg sodium per 100 kcal, 0.08% sodium on a dry matter basis] at 23:00 PM once daily for five days to attain a steady activation of RAAS^43^. The volume of the low-sodium diet was calculated so that the dogs received the same caloric intake as their regular diet.

On intensive sampling days (days 1, 18 and 35), blood was collected starting at 07:00 (0 h, immediately before oral benazepril dosing) and repeated at 0.5; 1; 2; 4; 8; 12; 12.5; 13; 14; 16; 20; and 24 h post-dosing. Benazepril [Nelio 5 mg chewable tablets, Ceva Sante Animale] was administered on intensive sampling days following 0-h blood sampling (all dose groups) and 12-h sampling (twice-daily dose groups only). Benazepril dose was calculated to the nearest 1.25 mg increment. Blood pressure and echocardiograms were performed on days 2, 19, and 36 after completing the 24-h post-dosing intensive sample collection. After data collection on days 2, 35, and 36, dogs began a 10-day wash-out period between treatment periods during which they consumed their regular diet [Royal Canin Beagle Adult, 110 mg sodium per 100 kcal] at approximately 09:00 daily.

### Data collection

Venous blood samples were collected from an external jugular or cephalic vein with a 1-inch, 20- or 22-gauge needle attached to a 6 ml syringe. On intensive sampling days, approximately 4 mL of whole blood was collected at each time point, with 2 mL transferred to an additive-free collection tube and 2 mL transferred to a lithium heparin tube containing 11.2 uL of dichlorvos prepared as a 6 mg/mL solution in acetonitrile. On baseline and sparse sampling days, approximately 2 mL of whole blood was collected and placed in an additive-free tube only. All samples were centrifuged at 3000 rpm for 15 min, after which plasma or serum was transferred into cryovials that were then stored at − 80 °C for later analysis.

Body weight was measured on sparse sampling days using the same digital scale throughout the study. That measure was used to calculate the dose of benazepril that the dogs would receive on the following intensive sampling day. Systolic BP was estimated using a Doppler device with the dog positioned in lateral recumbency using a 4 cm cuff placed over the left or right dorsal pedal artery. A minimum of five consistent BP measurements were obtained and averaged per session per dog. Transthoracic echocardiographic examinations were performed by the same board-certified cardiologist (JLW) using an ultrasound system coupled to a 5–8 MHz phased array sector transducer. Echocardiographic images were stored digitally and analyzed with an integrated image analysis system. Images were measured with digital calipers, and all measurements were averaged over five observations of sufficient technical quality.

### Bioanalytical methods for benazeprilat determination in plasma

Analysis of plasma benazeprilat (active metabolite of benazepril) was performed by the Iowa State University Analytical Chemistry Laboratory. Benazeprilat and benazeprilat-d5 stock standard solutions were prepared at 0.25 mg/mL in 2:1:1 acetonitrile:water:DMSO. Benazepril and benazepril-d5 stock solutions were prepared at 1 mg/mL in acetonitrile. A sample volume of 150 µL was fortified with 15 µL of a benazeprilat-d5 solution at 0.1 ppm. Plasma samples were precipitated with 600 µL of acetonitrile containing 0.5% formic acid and vortexed by hand. All samples were centrifuged at 10,000 rpm for 5 min. A 600 µL volume of each sample was transferred to a clean 2 mL microcentrifuge tube and concentrated to dryness. All samples were reconstituted in 100 µL of 50:50 acetonitrile: water and centrifuged at 10,000 rpm for 5 min prior to liquid chromatography-tandem mass spectrometry (LC–MS/MS) analysis. All samples were analyzed using an injection volume of 2 µL.

A Vanquish Flex LC pump interfaced with a TSQ Altis mass spectrometer [Thermo Fisher Scientific, San Jose, CA, USA] were used for the analysis. The source conditions were as follows: spray voltage—3500 V, sheath gas—40.6 Arb, auxiliary gas—23 Arb, sweep gas—0.4 Arb, ion transfer tube temperature − 325 °C, and vaporizer temperature − 350 °C. The chromatographic peak width was 2 s, and the cycle time was 0.2 s. The mass spectrometer was operated in positive ion electrospray ionization mode. Data were acquired using a multiple reaction monitoring method that selected for the benazeprilat ([M + H] + 397.2) and benazeprilat-d5 ([M + H] + 402.2) precursor ions.

The column used for the analysis was Hypersilgold Aq 50 × 2.1 mm, 1.9 µm [Thermo Fisher Scientific, Waltham, MA, USA]. Mobile Phase A was water + 0.1% formic acid, and Mobile Phase B was acetonitrile + 0.1% formic acid. The column oven temperature was set to 35 °C. The chromatography gradient was as follows: start at 0% B and linear ramp to 100%B in 2.0 min, hold at 100% B for 0.4 min, drop to 0% B in 0.01 min, and hold at 0% B for 0.59 min. The flow rate of the method was 0.4 mL/min.

### RAAS biomarker analysis

Equilibrium concentrations of AngI, AngII, AngIII, AngIV, Ang1–7, Ang1–5, and aldosterone were quantified in serum samples by LC–MS/MS performed at a commercial laboratory [Attoquant Diagnostics, Vienna, Austria] using previously validated and described methods^44–46^ following ex vivo equilibration. Briefly, samples were spiked with a stable isotope-labeled internal standard for each Ang peptide and a deuterated internal standard for aldosterone (aldosterone D4) after equilibration, and analytes were extracted using C18-based solid-phase extraction. Extracted samples were analyzed using mass spectrometry analysis using a reversed-analytical column operating in line with a XEVO TQ-S triple quadrupole mass spectrometer in multiple reaction monitoring mode. Internal standards were used to correct for analyte recovery across the sample preparation procedure in each individual sample. Analyte concentrations were calculated from integrated chromatograms considering the corresponding response factors determined in appropriate calibration curves in the serum matrix when integrated signals exceeded a signal-to-noise ratio of 10. Angiotensin-based markers for renin (PRA-S) and angiotensin-converting enzyme (ACE-S) were derived from AngII and AngI levels by calculating their sum and ratio, respectively^46,47^. The ratio of aldosterone/AngII (AA2-ratio) was calculated to assess adrenal responsiveness following AngII signaling resulting in the release of aldosterone^48^ as described by the analytical laboratory. Renin-independent alternative RAAS activation (ALT-S) was calculated using the formula [(Ang 1–7 + Ang 1–5)/(Ang I + Ang II + Ang 1–7 + Ang 1–5)]^49^.

### Statistical analysis

Statistical analyses were performed using commercial software [R version 4.2.1, R Foundation for Statistical Computing, Vienna, Austria]. Normality of the data was assessed using the Shapiro–Wilk test. Most data were non-normally distributed, so all data are reported as median (interquartile range). Outliers were included in all statistical analyses.

The area under the curve (AUC) of the benazeprilat concentration time-course was calculated for the first 12 h after dosing to assess the linearity in the dose-exposure response. Linear mixed modeling was used to determine the relationship between benazepril dose and exposure (AUC), with the individual dog being used as a random effect in the statistical model.

Blood pressure and echocardiographic variables were compared between dosing groups and baseline using either *t* tests (for normally distributed data) or Wilcoxon rank-sum tests (for non-normally distributed data).

The dose–response relationship of benazeprilat on the classical and alternative arm of the RAAS was characterized by the area under the effect curve of each RAAS biomarker after the first 12 h of dosing. Calculation of the 12-h time-weighted average (TWA) for each biomarker of interest (AngI, AngII, Ang1-7, Ang1-5, aldosterone, PRA-S, ACE-S, ALT-S, and AA2) was performed by dividing the area under the effect curve (determined using the trapezoidal rule) by the observation period (12 h), as previously described^50^. Time weighted averages were compared between dosing groups using Wilcoxon rank-sum tests, and the extent of the effect was assessed by calculating the percent change of TWA before and after dosing with oral benazepril. Finally, to assess any potential carryover effect on RAAS biomarkers, an analysis of variance (ANOVA) was performed on the sum of TWAs for each biomarker using Benjamini–Hochberg correction and comparing to the null hypothesis (no carryover).

## Results

### Systemic exposure to benazeprilat

There was no evidence of a carryover effect between dosing groups for any variable tested (adjusted *P*-values > 0.05 for all variables). The AUC for benazeprilat exposure over the first 12 h following a single oral dose of benazepril was significantly and positively related to dose amount (*P* < 0.001). A 1 mg increase in dose corresponded to an increase in AUC of 22.4 ng*h/L (95% confidence interval [CI] 13.3–31.5 ng*h/L; standard deviation 32.9 for random effect of dog). A twofold increase in benazepril dose corresponded to a 2.36-fold increase in AUC (95% CI 2.13 to 2.62; standard deviation 0.22 [in natural logarithmic scale] for random effect of dog).

### Clinical and hemodynamic variables

Clinical and hemodynamic data following each treatment are presented in Table 1, and results of group comparisons are provided in Table 2. Significant differences from baseline were noted at various benazepril dosages for the variables of heart rate, BP, fractional shortening, maximum velocity of late diastolic transmitral inflow (A vel), ratio of early to late diastolic transmitral inflow (E:A), isovolumic relaxation time (IVRT), and ratio of early diastolic transmitral inflow to IVRT (E:IVRT). The only significant differences between benazepril dosage groups occurred for the variables of heart rate and fractional shortening, and were only noted in the comparison between 0.25 and 0.5 mg/kg dosages. No significant differences from baseline or between dosage groups were noted for other clinical variables or echocardiographic indices.

**Table 1:** Clinical data at baseline and following oral benazepril dosing at 0.125, 0.25, or 0.5 mg/kg in 9 healthy beagle dogs.

Variable	Baseline	0.125 mg/kg	0.25 mg/kg	0.5 mg/kg
Weight (kg)	10.3 (9.9–11.0)	10.4(9.8–10.7)	10.1 (9.8–10.6)	10.0 (9.8–10.5)
Heart rate (bpm)	100 (90–110)	90(80–100)	100 (100–110)	80 (76–95)
BP (mmHg)	125 (120–136)	150 (144–152)	145 (140–160)	135 (129–156)
LVIDd (cm)	3.1 (2.9–3.2)	3.2 (3.1–3.2)	3.2 (3.0–3.4)	3.0 (3.0–3.1)
LVIDs (cm)	1.9 (1.8–2.0)	1.9 (1.8–2.0)	1.9 (1.6–1.9)	2.0 (1.7–2.0)
LA:Ao	1.1 (1.1–1.2)	1.1 (1.1–1.1)	1.2 (1.1–1.2)	1.1 (1.0–1.2)
FS (%)	37.8 (34.4–38.6)	39.8 (37.9–42.5)	45.5 (39.8–46.6)	38.4 (34.6–42.1)
EDVI	52.7 (49.1–60.9)	48.0 (40.3–52.9)	50.1 (44.4–52.5)	50.9 (47.8–57.8)
ESVI	16.7 (16.4–20.7)	16.9 (14.6–17.7)	16.4 (13.3–18.0)	18.8 (15.6–22.0)
EF (%)	66.5 (65.3–69.1)	65.2 (63.7–67.7)	68.7 (64.3–71.9)	62.1 (59.8–74.4)
E vel (m/s)	0.80 (0.75–0.93	0.75 (0.71–0.81)	0.77 (0.68–0.83)	0.73 (0.68–0.82)
A vel (m/s)	0.48 (0.44–0.54)	0.34 (0.28–0.34)	0.40 (0.37–0.42)	0.35 (0.28–0.40)
E:A	1.65 (1.53–1.80)	2.39 (2.02–2.86)	1.85 (1.72–2.19)	2.16 (1.93–2.61)
IVRT (ms)	61 (58–61)	70 (67–74)	63 (61–74)	67 (64–72)
E:IVRT	1.42 (1.28–1.48)	1.13 (1.05–1.22)	1.12 (0.98–1.23)	1.07 (0.98–1.21)

Data are presented as median (interquartile range).

*BP* blood pressure, *LVIDd* left ventricular internal diameter in diastole, *LVIDs* left ventricular internal diameter in systole, *LA:Ao* ratio of left atrium to aorta diameter, *FS* fractional shortening, *EDVI* end-diastolic volume index, *ESVI* end-systolic volume index, *EF* ejection fraction, *E vel* maximum velocity of early diastolic transmitral inflow, *A vel* maximum velocity of late diastolic transmitral inflow, *E:A* ratio of early to late diastolic transmitral inflow, *IVRT* isovolumic relaxation time, *E:IVRT* ratio of early diastolic transmitral inflow to IVRT.

**Table 2:** P-values for group comparisons for clinical and hemodynamic variables at baseline vs. following oral benazepril dosing at 0.125, 0.25, or 0.5 mg/kg in 9 healthy beagle dogs.

Variable	Baseline vs. 0.125 mg/kg	Baseline vs. 0.25 mg/kg	Baseline vs. 0.5 mg/kg	0.125 vs. 0.25 mg/kg	0.25 vs. 0.5 mg/kg	0.125 vs. 0.5 mg/kg
Weight	0.83	0.54	0.54	0.79	0.93	0.89
Heart rate	0.074	0.64	0.011*	0.18	0.034*	0.40
BP	0.010*	0.044*	0.14	0.54	0.57	0.24
LVIDd	0.33	0.27	1.00	0.72	0.19	0.22
LVIDs	0.87	0.40	0.69	0.50	0.66	0.81
LA:Ao	0.42	0.53	0.42	0.16	0.16	1.00
FS	0.57	**0.033**	0.88	0.11	0.046*	0.68
EDVI	0.16	0.26	0.48	0.67	0.72	0.66
ESVI	0.55	0.21	0.78	0.51	0.13	0.39
EF	0.34	0.83	0.34	0.35	0.22	0.45
E vel	0.22	0.12	0.20	0.73	0.78	0.95
A vel	0.00053*	0.013*	0.0018*	0.23	0.43	0.66
E:A	0.015*	0.10	0.0054*	0.26	0.39	0.73
IVRT	0.034*	0.044*	0.063	0.91	0.86	0.77
E:IVRT	0.024*	0.031*	0.057	0.80	0.96	0.72

Group comparisons were performed using *t* tests (normally-distributed data) or Wilcoxon rank-sum tests (non-normally distributed data). Significant differences (P-value < 0.05) are designated with bolding and an asterisk (*).

*BP* blood pressure, *LVIDd* left ventricular internal diameter in diastole, *LVIDs* left ventricular internal diameter in systole, *LA:Ao* a ratio of left atrium to aorta diameter, *FS* fractional shortening, *EDVI* end-diastolic volume index, *ESVI* end-systolic volume index, *EF* ejection fraction, *E vel* maximum velocity of early diastolic transmitral inflow, *A vel* maximum velocity of late diastolic transmitral inflow, *E:A* ratio of early to late diastolic transmitral inflow, *IVRT* isovolumic relaxation time, *E:IVRT* ratio of early diastolic transmitral inflow to IVRT.

### RAAS biomarkers

**Figure 3:**
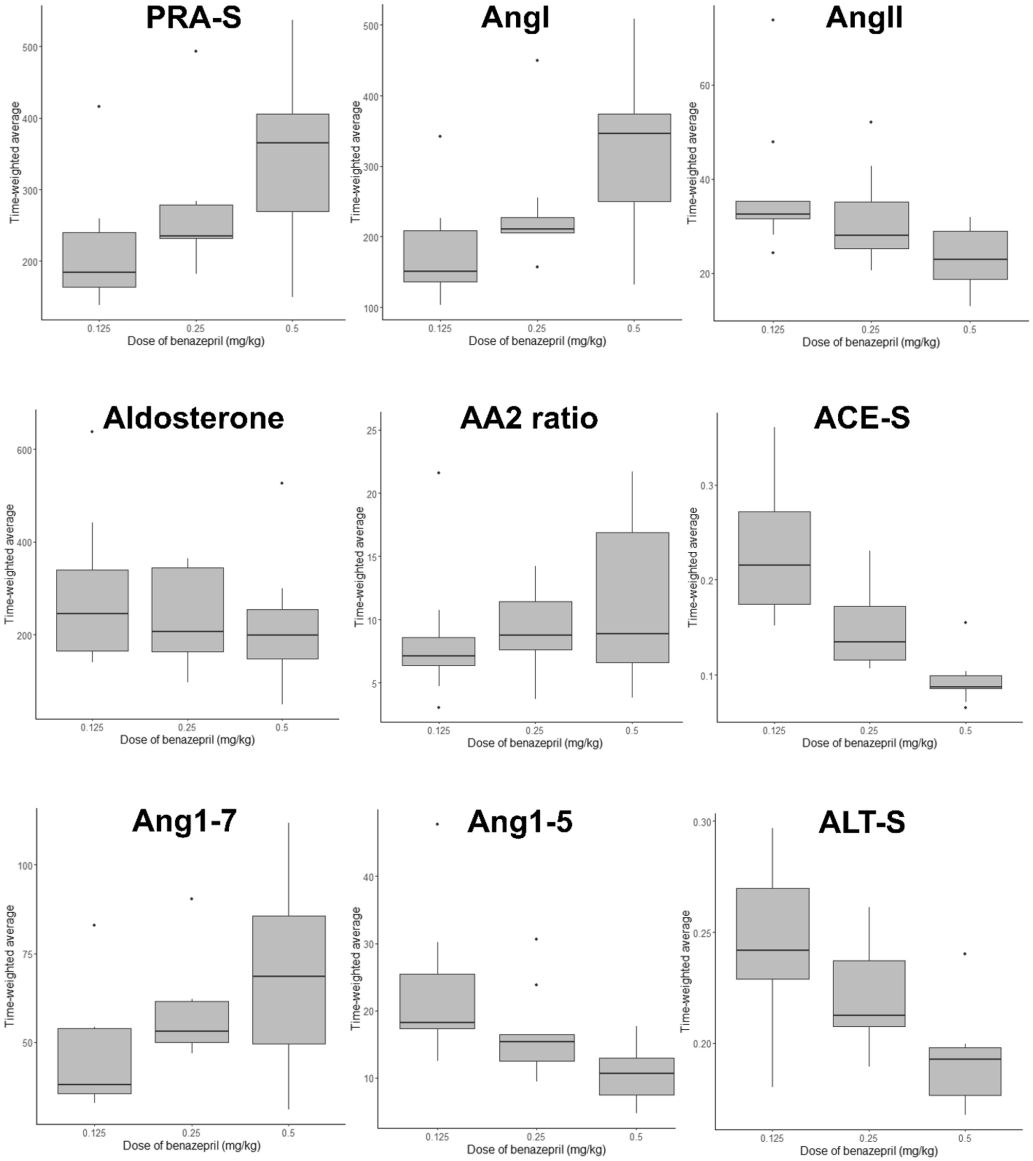
Twelve hour time-weighted average of RAAS biomarkers in nine healthy beagle dogs treated with a single dose of benazepril at 0.125, 0.25, or 0.5 mg/kg orally. Units for time-weighted averages of biomarkers are pmol/L; ratios are unitless. The horizontal line represents median, box represents quartiles, and whiskers represent range, with outliers plotted as dots. Outliers are defined as point occurring > 1.5 × IQR higher than Q3 or lower than Q1. *Ang* angiotensin, *PRA*-S surrogate measure of plasma renin activity, *ACE*-S surrogate measure of angiotensin-converting enzyme activity, ALT-S surrogate measure of alternative RAAS pathway activity, *AA2 ratio* measure of adrenal responsiveness to angiotensin II.

**Figure 4:**
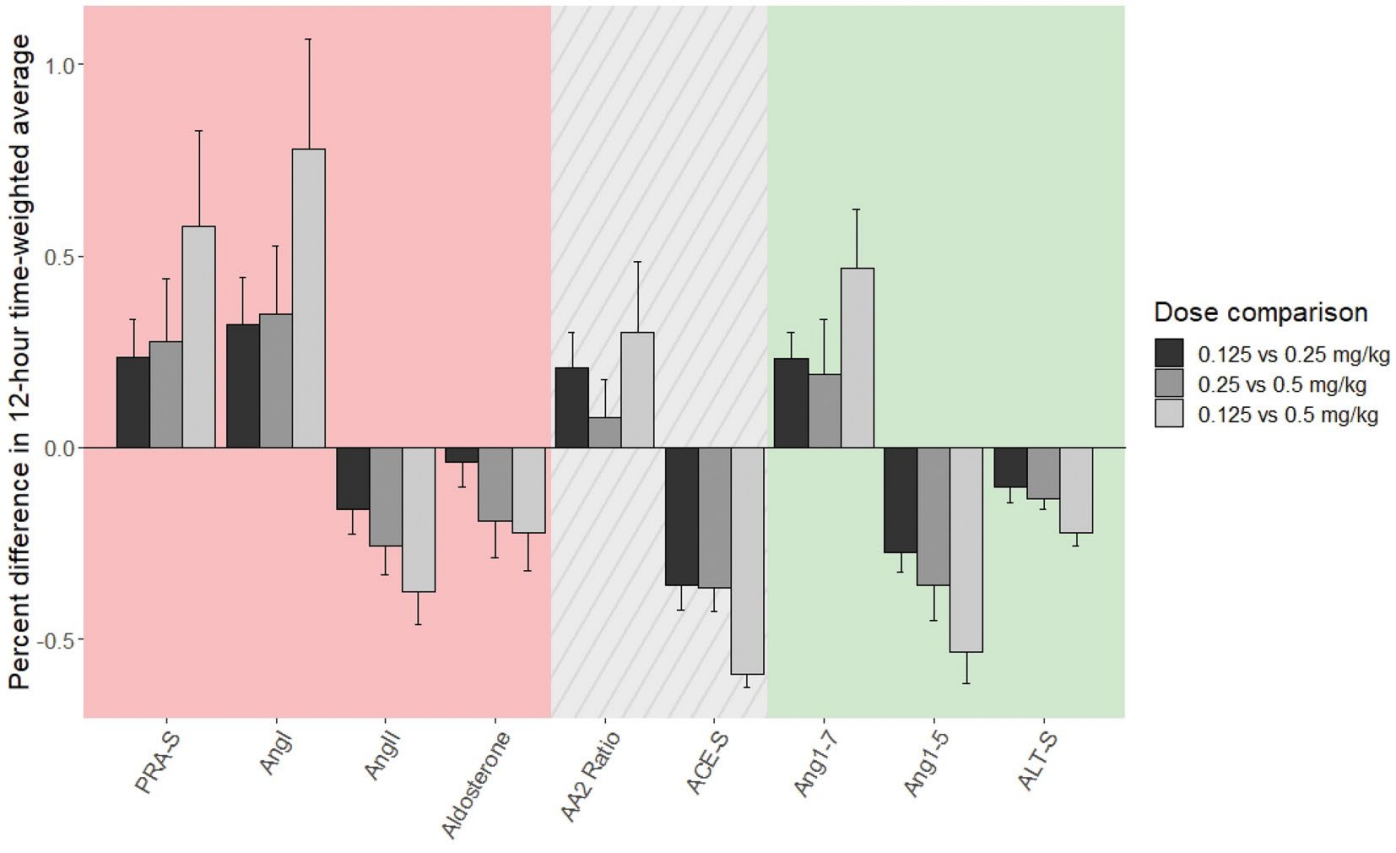
Comparison of treatment effect size (percent difference in 12-h time-weighted averages) on RAAS biomarkers in nine healthy beagle dogs treated with a single oral dose of benazepril at 0.125, 0.25, or 0.5 mg/kg. The extent of treatment effect for each dosage comparison is calculated as (time-weighted average for dosage B – time-weighted average for dosage A)/(time-weighted average for dosage B). Positive bars indicate *higher* values of the indicated biomarker for the higher benazepril dosage (corresponding to positive values for the extent of treatment effect in Table 4). In comparison, negative bars indicate *lower* values of the indicated biomarker with the higher benazepril dosage (corresponding to negative values for the extent of treatment effect in Table 4). Error bars represent the standard error of treatment effect size. Red shading indicates activity of the classical RAAS; green shading indicates activity of the alternative RAAS; grey shading with diagonal lines indicates other ratios. *Ang* angiotensin, *PRA-S* surrogate measure of plasma renin activity, *ACE-S* surrogate measure of angiotensin-converting enzyme activity, *ALT-S* surrogate measure of alternative RAAS pathway activity, *AA2 ratio* measure of adrenal responsiveness to angiotensin II.

For RAAS biomarkers, the 12-h TWA for the different dosing groups is displayed in Table 3 and Fig. 3. Comparisons between dosing groups for these RAAS biomarkers, including the extent of treatment effect size and *P*-values for TWA comparisons among groups, are presented in Table 4. Comparison of treatment effect size is further displayed graphically in Fig. 4. Significant differences in ACE-S (a surrogate for ACE activity) were noted for all dosage comparisons. For the comparison of 0.125 and 0.5 mg/kg dosage groups, significant differences were also noted for PRA-S (surrogate for plasma renin activity), AngI, AngII, Ang1-5, and ALT-S (surrogate for alternative RAAS activation). For the comparison of 0.25 and 0.5 mg/kg dosage groups, a significant difference was also noted for ALT-S.

**Table 3:** Twelve hour time-weighted average of RAAS biomarkers in nine healthy beagle dogs treated with a single dose of benazepril at 0.125, 0.25, or 0.5 mg/kg orally.

Variable	0.125 mg/kg	0.25 mg/kg	0.5 mg/kg
PRA-S	184.3 (163.5–240.5)	234.9 (231.8–279.1)	364.9 (269.5–406.0)
AngI	151.0 (136.4–209.0)	210.8 (205.7–227.0)	346.2 (250.2–374.1)
AngII	32.52 (31.55–35.36)	28.00 (25.16–35.12)	22.81 (18.70–28.95)
Aldosterone	244.3 (164.5–338.6)	206.3 (163.8–343.9)	198.3 (148.5–253.5)
AA2 ratio	7.13 (6.39–8.60)	8.79 (7.63–11.44)	8.86 (6.60–16.88)
ACE-S	0.22 (0.17–0.27)	0.13 (0.12–0.17)	0.09 (0.09–0.10)
Ang1-7	37.93 (35.53–53.91)	52.96 (49.92–61.41)	68.52 (49.62–85.64)
Ang1-5	18.24 (17.32–25.43)	15.39 (12.51–16.43)	10.67 (7.42–12.97)
ALT-S	0.24 (0.23–0.27)	0.21 (0.21–0.24)	0.19 (0.18–0.20)

Units for time-weighted averages of biomarkers are pmol/L; ratios are unitless. Data are presented as median (interquartile range).

*Ang* angiotensin, *PRA-S* surrogate measure of plasma renin activity, *ACE-S* surrogate measure of angiotensin-converting enzyme activity, *ALT-S* surrogate measure of alternative RAAS pathway activity, *AA2* ratio measure of adrenal responsiveness to angiotensin II.

**Table 4:** Extent of treatment effect (percent difference between dosages) and P-values for group comparisons of time-weighted average of RAAS biomarkers during the first 12 h after oral benazepril dosing.

Dosage	0.125 vs. 0.25 mg/kg		0.25 vs. 0.5 mg/kg		0.125 vs. 0.5 mg/kg	
Variable	Extent of treatment effect	P-value	Extent of treatment effect	P-value	Extent of treatment effect	P-value
PRA-S	0.24 (0.07 to 0.42)	0.11	0.28 (− 0.07 to 0.55)	0.19	0.58 (0.10 − 1.10)	0.040*
AngI	0.32 (0.1 to 0.53)	0.05	0.35 (− 0.05 to 0.65)	0.16	0.78 (0.26 to 1.49)	0.014*
AngII	− 0.16 (− 0.29 to 0.08)	0.26	− 0.26 (− 0.32 to − 0.06)	0.14	− 0.38 (− 0.50 to − 0.30)	0.004*
Aldosterone	− 0.04 (− 0.16 to 0.08)	1.00	− 0.19 (− 0.26 to − 0.18)	0.60	− 0.22 (− 0.41 to − 0.13)	0.55
AA2 Ratio	0.21 (0.07 to 0.33)	0.39	0.08 (− 0.002 to 0.19)	0.66	0.30 (− 0.004 to 0.37)	0.34
ACE-S	− 0.36 (− 0.49 to − 0.19)	0.014*	− 0.36 (− 0.51 to − 0.19)	0.0008*	− 0.59 (− 0.62 to − 0.54)	0.0002*
Ang1-7	0.23 (0.15 to 0.43)	0.11	0.19 (− 0.06 to 0.52)	0.60	0.47 (0.24 to 0.57)	0.09
Ang1-5	− 0.27 (− 0.35 to − 0.15)	0.09	− 0.36 (− 0.48 to − 0.21)	0.06	− 0.53 (− 0.65 to − 0.42)	0.001*
ALT-S	− 0.10 (− 0.15 to − 0.05)	0.16	− 0.14 (− 0.2 to − 0.08)	0.02*	− 0.22 (− 0.24 to − 0.18)	0.004*

Extent of treatment effect (percent change) for each dosage comparison is calculated as: (TWA for dosage B – TWA for dosage A)/(TWA for dosage B). Data are presented as median (IQR). Positive treatment effect size indicates *higher* values of the indicated biomarker for the higher benazepril dosage, while negative treatment effect size indicates *lower* values of the indicated biomarker with the higher benazepril dosage. Group comparisons of 12-h TWAs were performed using Wilcoxon rank-sum tests. Significant differences (P-value < 0.05) are designated with bolding and an asterisk (*).

*Ang* angiotensin, *PRA-S* surrogate measure of plasma renin activity, *ACE-S* surrogate measure of angiotensin-converting enzyme activity, *ALT-S* surrogate measure of alternative RAAS pathway activity, *AA2 ratio* measure of adrenal responsiveness to angiotensin II.

## Discussion

This study is the first to assess a dose–response relationship of an ACEI on the comprehensive RAAS fingerprint and hemodynamic variables in dogs. Overall, our results demonstrated dose-dependent effects of benazepril on a subset of RAAS biomarkers from both classical and alternative pathways. Specifically, compared to the lowest dosage (0.125 mg/kg), the highest dosage (0.5 mg/kg) of benazepril was associated lower values of ACE-S (− 59%), AngII (− 38%), Ang1-5 (− 53%), and ALT-S (− 22%), and higher values of AngI (+ 78%) and PRA-S (+ 58%). These findings are logical given the mechanism of ACEI in the context of both classical and alternative RAAS pathways: inhibition of ACE will lead to higher levels of substrates (AngI and Ang1-7) and lower levels of products (AngII and Ang1-5) for the reactions catalyzed by this enzyme^41^. Except for the ratios ACE-S and ALT-S (which amplify these substrate/product comparisons), significant differences between dosage groups were noted only for the comparison between the lowest and highest dosage utilized, suggesting that small differences in ACEI dosage might have minimal impact on RAAS biomarkers.

This study has several strengths compared to previous assessments of ACEI pharmacodynamics in dogs. First, the experimental method of RAAS activation used in this study (a low-sodium diet) has been shown to provide a more steady and reliable activation of RAAS compared to the use of pharmacologic agents such as furosemide or amlodipine^43^. Second, this study utilized a comprehensive RAAS fingerprint technique to analyze multiple RAAS biomarkers simultaneously, rather than using surrogates of RAAS activity (ACE activity or urinary aldosterone concentration) that do not accurately reflect RAAS biomarker concentrations^19–22,51^. A particular benefit of the comprehensive RAAS fingerprint lies in its ability to compare activity of the classical and alternative RAAS pathways simultaneously. Greater ACE inhibition should theoretically provide more substrate for the alternative pathway, leading to favorable rebalancing of alternative vs. classical RAAS^41^. However, the ultimate biologic effect of RAAS is determined not by circulating concentrations of biomarkers but the interaction of biomarkers with their target receptors. For example, while AngII binding to angiotensin type I receptors causes vasoconstriction and sodium retention (biological effects associated with “classical” RAAS), binding of AngII to angiotensin type 2 receptors leads to counterregulatory vasodilatory and natriuretic effects^52^. While our results suggest a more favorable profile of RAAS peptides with higher doses of benazepril (in particular, a 38% reduction in AngII with benazepril 0.5 mg/kg compared to 0.125 mg/kg), the clinical benefit of these differences in RAAS biomarkers cannot be inferred from our results. Nonetheless, our results underscore the importance of looking beyond single surrogates of RAAS activity (such as ACE activity or aldosterone) utilized in previous pharmacodynamic studies, as these values may be poorly correlated with concentrations of Ang peptides and do not reflect the complexity of classical versus alternative RAAS activity^16–18^.

A secondary aim of this study was to investigate the dose–response relationship of benazepril on BP and echocardiographic measurements. Blood pressure was actually *higher* following all benazepril dosages compared to baseline, likely due to the stress of 24-h intensive blood sampling performed before BP in all treatment groups (which did not occur prior to baseline BP measurement). This was not surprising since the effect of environmental stress on noninvasive BP measurements in dogs is well-documented^53^. Since BP timing was consistent with respect to procedures in all treatment groups, we were able to compare BP (and change from baseline in BP) between dosage groups to look for evidence of a dose-dependent effect in mitigating the stress-induced increase in BP; differences were not statistically significant. This is not surprising given that the subjects of this study were healthy dogs with intact mechanisms for renal regulation of BP. Furthermore, previous literature in dogs with renal or cardiac disease showed only modest and variable decrease in BP following repeated ACEI administration in dogs, generally in the range of 5–15 mmHg^29,31,32,54^.

Echocardiographic variables were largely unaffected by benazepril administration at any dosage. This is consistent with the drug's mechanism of action; ACEI would not be expected to cause significant cardiac effects in patients without local cardiac tissue RAAS activation, particularly in a short-term setting. Our findings are also consistent with a previous study of the short-term hemodynamic effects of enalapril in dogs with naturally-occurring CHF, which reported no change in echocardiographic indices of left ventricular size and function compared to placebo after 2 or 21 days of enalapril treatment^34^. In other clinical trials of dogs with naturally-occurring CHF that compare effects of ACEI to placebo^3,55^, results of serial echocardiographic examinations post-treatment were not reported. In our study, echocardiographic variables that differed from baseline following benazepril treatment were primarily those associated with left ventricular diastolic function (relaxation and filling), including A vel, IVRT, and ratios that include these variables (E:A and E:IVRT). While reduction in A vel is challenging to interpret in isolation and may not be clinically relevant, E:IVRT has been reported as an echocardiographic predictor of left atrial pressure in several experimental studies^56–59^. It is theoretically possible that benazepril led to a mild decrease in left atrial pressure in this study, as has been previously reported in dogs with experimental chordae tendineae rupture treated with the ACEI alacepril^31^. However, it is challenging to interpret the clinical relevance of changes in E:IVRT with no change in E wave velocity, and the changes from baseline in diastolic variables in this study were small and not dose-dependent. Overall, the results of this study are consistent with previous reports that ACEI do not cause clinically important echocardiographic changes in healthy dogs. Given the extent of benazepril’s treatment effect on RAAS biomarkers in this experimental model of RAAS activation, this also underscores the insensitivity of echocardiography to detect changes in RAAS biomarker profiles, particularly in healthy dogs.

This study had several limitations. A placebo-treated control group was not included, so changes in RAAS biomarkers are interpreted as differences from baseline or differences between dose groups. Time-weighted average of RAAS biomarkers in this study was limited to the first 12 h after a single dose of benazepril, and we are unable to comment on the effects of repeated dosing or compare twice daily versus once daily dosing. Due to wide variability for some RAAS biomarkers and small group size in our study (both of which increase risk of type II error), we chose to report non-adjusted P-values from group comparisons and focus on the extent of treatment effect to best represent the biological relevance of our results^60,61^. Echocardiography and noninvasive (Doppler) BP are insensitive tools for detecting subtle hemodynamic changes, particularly in dogs without pre-existing cardiovascular disease. Our study subjects were clinically healthy dogs with experimentally-induced RAAS activation; the effects of benazepril on RAAS biomarkers, BP, and echocardiography might be different in dogs with naturally-occurring heart disease.

## Conclusion

Benazepril has dose-dependent but nonlinear effects on several biomarkers of the classical and alternative RAAS in healthy dogs with experimentally-induced RAAS activation. In particular, levels of AngII were 38% lower and levels of Ang1-7 were 47% higher in dogs receiving benazepril at a dosage of 0.5 mg/kg compared to 0.125 mg/kg. These data represent the first attempt to characterize how ACEI dosage might impact biomarkers from both classical and alternative RAAS pathways, and may have useful translational impact for dosage optimization studies in humans receiving ACEI. Given the inherent limitations of single-dose pharmacokinetic/pharmacodynamic evaluations and the complexity of the biological activity of the RAAS, further studies are needed to inform dosage recommendations for benazepril in dogs.

## Data Availability

The data generated during this study are available from the corresponding author on reasonable request.
